# Proteomics of Maize Root Development

**DOI:** 10.3389/fpls.2018.00143

**Published:** 2018-03-05

**Authors:** Frank Hochholdinger, Caroline Marcon, Jutta A. Baldauf, Peng Yu, Felix P. Frey

**Affiliations:** Crop Functional Genomics, Institute of Crop Science and Resource Conservation, University of Bonn, Bonn, Germany

**Keywords:** heterosis, lateral root, maize, proteomics, primary root, root hairs, seminal root, shoot-borne root

## Abstract

Maize forms a complex root system with structurally and functionally diverse root types that are formed at different developmental stages to extract water and mineral nutrients from soil. In recent years proteomics has been intensively applied to identify proteins involved in shaping the three-dimensional architecture and regulating the function of the maize root system. With the help of developmental mutants, proteomic changes during the initiation and emergence of shoot-borne, lateral and seminal roots have been examined. Furthermore, root hairs were surveyed to understand the proteomic changes during the elongation of these single cell type structures. In addition, primary roots have been used to study developmental changes of the proteome but also to investigate the proteomes of distinct tissues such as the meristematic zone, the elongation zone as well as stele and cortex of the differentiation zone. Moreover, subcellular fractions of the primary root including cell walls, plasma membranes and secreted mucilage have been analyzed. Finally, the superior vigor of hybrid seedling roots compared to their parental inbred lines was studied on the proteome level. In summary, these studies provide novel insights into the complex proteomic interactions of the elaborate maize root system during development.

## Introduction

**FIGURE 1:**
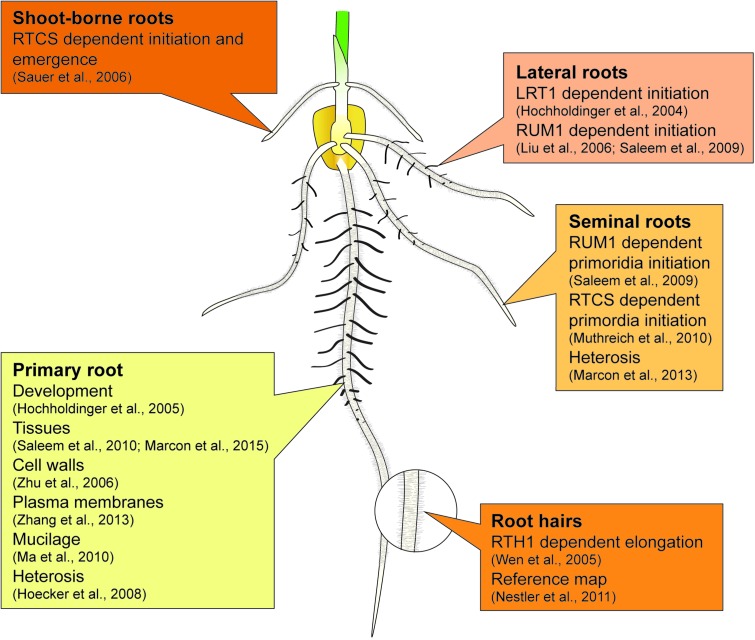
Summary of proteome studies related to individual root types of maize.

Maize forms a complex root system to capture limited and unevenly distributed water and nutrient resources from soil and allocating them to the energy-delivering, aboveground parts of the plant. The basic blueprint of the elaborate three-dimensional structure of the maize root system is encoded in an intrinsic genetic program, while the responsiveness to environmental signals secures the adaptive plasticity of the maize root stock (Hochholdinger et al., 2004a,b, 2017). The maize root system is composed of primary and seminal roots formed during embryogenesis and of shoot-borne and lateral roots initiated post-embryonically after germination (**Figure 1**) (Hochholdinger et al., 2004a; Hochholdinger, 2009; Atkinson et al., 2014). The primary root and a variable number of seminal roots are already preformed during embryogenesis. After germination, these roots are important for early seedling vigor (Sanguineti et al., 1998). Whorls of shoot-borne roots are initiated at successive nodes of the shoot and dominate the rootstock of mature plants (Hochholdinger et al., 2004b). Finally, lateral roots are formed in all root types by the meristematic activity of phloem pole pericycle cells. Together with root hairs, which are unicellular extensions of epidermal cells, lateral roots substantially increase the absorbing surface of the maize root system (Yu et al., 2016).

Large-scale quantitative approaches can provide a comprehensive snapshot of biologically relevant molecules such as transcripts, metabolites or proteins in a biological sample. While the transcriptomic dissection of maize root development has been recently reviewed elsewhere (e.g., Yu et al., 2016; Hochholdinger et al., 2017), here we will give an update on the status of the proteomic dissection of maize root development. The proteome is defined as the protein complement of a given biological sample, while proteomics refers to the global identification of these proteins (Wilkins et al., 1995). Initial proteomic studies in maize (Chang et al., 2000; Porubleva et al., 2001) were based on the two-dimensional electrophoretic (2-DE) separation of protein extracts and the subsequent in-gel visualization of distinct protein spots by a variety of staining techniques (Hochholdinger et al., 2006). Proteins separated on 2-DE gels were then individually excised, proteolytically digested and subjected to mass spectrometry. More recently, protein samples are directly subjected to a shotgun mass spectrometric analysis without prior separation (e.g., Marcon et al., 2015). During mass spectrometry, peptides are ionized by electrospray ionization (ESI) or matrix-assisted laser desorption ionization (MALDI). Subsequently, these peptides are separated in the gas phase, according to their mass-to-charge (m/z) ratio in a mass analyzer and recorded with a detector. Finally, the peptides are identified by comparing the detected mass spectra with theoretical spectra generated from *in silico* digestions of protein databases. Details on the mass spectrometric analysis of proteins have been reviewed elsewhere (e.g., Chait, 2011).

The focus of this review is to highlight the status of the proteomic dissection of processes involved in the endogenous genetic control of maize root development. Endogenous factors that alter the proteome of maize roots such as the availability of mineral nutrients (e.g., Li et al., 2015) or the influence of abiotic stress (e.g., Ghosh and Xu, 2014; Ghatak et al., 2017) will not be discussed.

## Proteomics of Maize Root System Architecture

### Primary Root

The single primary root is preformed in the embryo and emerges from the basal pole of the seed 2–3 days after germination (Hochholdinger et al., 2004a). It has been demonstrated that the majority of the soluble proteome of maize primary roots of the inbred line B73 significantly changes between 5 and 9 days after germination. Only 28% of proteins identified in 5-day-old primary roots were also observed in 9-day-old primary roots (Hochholdinger et al., 2005).

In longitudinal orientation, maize roots are organized in several partially overlapping zones of development comprising the meristematic, elongation and differentiation zones (Ishikawa and Evans, 1995). To study the molecular function of radial zones of development, the differentiation zone of 2.5-day-old primary roots was manually separated into cortical parenchyma and stele (Saleem et al., 2010). The stele comprises the central vascular cylinder and the pericycle, while the cortical parenchyma includes all tissues surrounding the central vascular cylinder. The cortical parenchyma comprises a single endodermal cell layer, several layers of cortex cells and a single epidermal cell file. The stele transports water, nutrients and photosynthates, while the cortical parenchyma represents the ground tissue of the root, which has metabolic functions but is also involved in the transport of material into the stele. Phytohormone profiling revealed enhanced levels of auxin in the stele and of cytokinin in the cortical parenchyma (Saleem et al., 2010). Several cytokinin-dependent proteins were upregulated in the cortical parenchyma. This localization is consistent with the preferential accumulation of cytokinin in this tissue. Some of these proteins are enzymes, including a β-glucosidase that hydrolyze the cytokinin *cis*-zeatin *O*-glucoside and four enzymes involved in ammonium assimilation (Saleem et al., 2010). The antagonistic levels of auxin and cytokinin in the stele and cortical parenchyma and the cortical parenchyma-specific accumulation of cytokinin-regulated proteins highlight a molecular framework that regulates the function of these distinct root tissues (Saleem et al., 2010). Recently, a high-resolution map of the proteome and phosphoproteome of different tissues of the maize primary root (Marcon et al., 2015) complemented this initial tissue specific proteomic dissection of the maize primary root. In this analysis cortical parenchyma, stele, meristematic zone, and elongation zone were separately subjected to high-performance liquid chromatography coupled with tandem mass spectrometry. As a result, 11,552 distinct non-modified and 2,852 phosphorylated proteins were identified in these four root tissues (Marcon et al., 2015). Along the longitudinal axis, the abundance of functional protein classes was highlighted by two gradients. Along these gradients, the functional classes “RNA”, “DNA”, and “protein” peaked in the meristematic zone, whereas the categories “cell wall”, “lipid metabolism”, “stress”, “transport”, and “secondary metabolism” displayed maximum accumulation in the differentiation zone (Marcon et al., 2015). Comparison of this primary root tissue data set with high-resolution datasets of maize seed (Walley et al., 2013) and leaf (Facette et al., 2013) proteomes revealed that 13% of the identified proteins were exclusively detected in the primary root. These root-specific proteins displayed a high degree of tissue specific functionalization and underscored the plasticity of tissue specific proteomes in maize primary roots (Marcon et al., 2015).

On the subcellular level, the maize primary root was also a model to study the proteomes associated with cell walls (Zhu et al., 2007) and plasma membranes (Zhang et al., 2013). Cell wall proteins have been studied in the elongation zone of maize primary roots (Zhu et al., 2007). Among the identified cell wall proteins, many were involved in cell elongation and cell wall metabolism. Maize forms type II cell walls (Carpita, 1996). Therefore, several of the cell wall proteins identified in the elongation zone of maize primary roots have not been identified in proteomic studies that have focused on type I walls (Zhu et al., 2007). Similarly, the distribution of plasma membrane proteins in the growth zone of the maize primary root was studied during development (Zhang et al., 2013). The plasma membrane is the interface between the plant cell and the extracellular space or the apoplast and thus involved in the exchange of cellular molecules and in signal integration. This study demonstrated that cellulose synthases became more abundant with increasing distance from the root apex. This is consistent with the expected localization of cell wall deposition (Zhang et al., 2013).

Among other compounds, maize root cap cells secrete proteins into the rhizosphere via an amorphous gel structure called mucilage. From the mucilage secreted by 3 to 4-day-old primary roots 2,848 distinct extracellular proteins were identified by nanoLC–MS/MS (Ma et al., 2010). Among those, metabolic proteins represented the largest class. A comparison with the mucilage proteins previously identified in several dicot species suggested a considerable overlap between monocot and dicot mucilage proteomes (Ma et al., 2010).

### Seminal Roots

Seminal roots are the second embryonic root type of maize formed after the primary root. They emerge from the scutellar node and their number is variable between different genotypes but also within a given genotype (Hochholdinger et al., 2004a). The maize mutants *rtcs* (*rootless concerning crown and seminal roots*; Hetz et al., 1996) and *rum1* (*rootless with undetectable meristems 1*; Woll et al., 2005) do not initiate seminal roots. Both genes encode key components of auxin signal transduction. Auxin triggers transcriptional responses via the regulation of the activity of AUXIN RESPONSE FACTOR (ARF) proteins (reviewed in, Lavy and Estelle, 2016). At low auxin levels, Auxin/INDOLE-3-ACETIC ACID (Aux/IAA) transcriptional repressors interact with ARFs and repress their activity. In contrast, at high auxin levels TRANSPORT INHIBITOR RESPONSE 1/AUXIN SIGNALING F-BOX PROTEIN (TIR1/AFB) proteins bind to Aux/IAA transcriptional repressors and mediate their degradation via the proteasome. As a consequence, ARF transcription factors activate direct downstream target genes such as LOB domain transcription factors by binding to auxin response elements in their promoter (Xu et al., 2016). While *rtcs* encodes a LOB domain transcription factor (Taramino et al., 2007; Xu et al., 2015, 2016), *rum1* encodes an Aux/IAA transcriptional regulator (von Behrens et al., 2011). It was demonstrated that *rtcs* and *rum1* are not only critical for seminal root initiation but also for seminal root number because they likely underlie the two major QTLs controlling this trait (Salvi et al., 2016). Immature wild-type and *rtcs* embryos were subjected to a comparative proteome analysis 25 days after pollination (Muthreich et al., 2010). At this stage wild-type embryos started the initiation of seminal roots while mutant embryos did not. Among the differentially accumulated proteins, two phosphoglycerate kinases and a malate dehydrogenase, which are crucial checkpoints of cellular energetics, were preferentially accumulated in wild-type versus mutant embryos (Muthreich et al., 2010). Similarly, the proteomes of wild-type and *rum1* embryos were compared 30 days after pollination (Saleem et al., 2009). In maize, GLOBULIN 1 and GLOBULIN 2 are the most prevalent storage proteins in embryos (Kriz, 1989; Belanger and Kriz, 1991). These proteins are exclusively expressed during embryo development (Belanger and Kriz, 1991; Kriz, 1999). In total, seven GLOBULIN 1 isoforms and four GLOBULIN 2 isoforms were differentially accumulated between wild-type and *rum1* embryos 30 days after pollination suggesting significant regulation of these proteins by RUM1 (Saleem et al., 2009). It was pointed out that the regulation of GLOBULIN1 by the phytohormone abscisic acid (ABA), which stimulates root elongation and branching, might link this class of proteins to root formation (Saleem et al., 2009). Only little overlap was observed between embryonic proteins differentially accumulated between wild-type and *rtcs* embryos 25 days after pollination (Muthreich et al., 2010) and wild-type and *rum1* embryos 30 days after pollination (Saleem et al., 2009). This highlights the distinct regulation of the maize embryo proteome by RTCS and RUM1, but this difference could also be in part due to the analysis of the distinct stages of embryo development.

### Shoot-Borne Roots

After germination, whorls of shoot-borne roots are formed at successive nodal structures of the shoot (Hochholdinger et al., 2004b). The maize rootstock develops ∼70 shoot-borne roots in the course of development (Hoppe et al., 1986). Therefore, shoot-borne roots make up the major proportion of the adult maize root system. The previously described maize mutant *rtcs* (Hetz et al., 1996) is impaired in the initiation of all shoot-borne roots. In wild-type maize, the first node from which shoot-borne roots emerge is the coleoptilar node. In a proteome study, the soluble proteomes of wild-type versus mutant *rtcs* coleoptilar nodes were compared 5 and 10 days after germination (Sauer et al., 2006). These stages coincided with the initiation and emergence of shoot-borne roots in wild-type coleoptilar nodes, respectively. Several of the differentially accumulated proteins detected in this study are involved in the regulation of plant development (Sauer et al., 2006). The differentially accumulated proteins at the two developmental stages displayed only little overlap, indicating that distinct sets of proteins control initiation and emergence of lateral roots. RNA gel blot experiments of a subset of differentially accumulated proteins demonstrated that these expression differences are already manifested on the RNA level (Sauer et al., 2006).

### Lateral Roots

After germination, lateral roots emerge by division of pericycle cells in the differentiation zone of all root types (Hochholdinger et al., 2004b). The maize mutants *lrt1* (*lateral rootless 1*; Hochholdinger and Feix, 1998) and *rum1* (Woll et al., 2005) are impaired in the initiation of lateral roots. Differentially accumulated proteins were determined in a proteome survey of 9-day-old wild-type primary roots with lateral roots and *lrt1* primary roots without lateral roots (Hochholdinger et al., 2004c). Remarkably, 10% of proteins identified in this study were predominantly accumulated in *lrt1* primary roots that lack lateral roots suggesting that the presence of lateral roots can significantly influence the proteome of the primary root (Hochholdinger et al., 2004c). To study the molecular processes prior to lateral root formation, 2.5-day-old wild-type and mutant *rum1* primary roots were subjected to a proteome analysis (Liu et al., 2006). At this early stage of development no morphological differences were observed between the primary roots of the two genotypes. Nevertheless, differentially accumulated proteins involved in defense, lignin biosynthesis and in the citrate cycle were identified (Liu et al., 2006). In a follow up study the differentiation zone of 2.5-day-old wild-type and mutant *rum1* primary roots were manually separated in cortical parenchyma and stele and subjected to a proteome analysis (Saleem et al., 2009). Differentially accumulated proteins between the mutant *rum1* and wild-type demonstrated that RUM1 regulates the proteome of cortical parenchyma and stele tissues. Among the biochemical pathways regulated by RUM1, enzymes related to glycolysis were differentially expressed between these tissues (Saleem et al., 2009). It has been demonstrated that key enzymes of glycolysis such as hexokinase (Halford and Paul, 2003) and pyruvate kinase (Moore et al., 2003) control root elongation via sugar sensing and signaling. It was therefore suggested that the reduced primary root length of the *rum1* mutant could be conferred by tissue specific imbalances of glycolytic enzymes during root development (Saleem et al., 2009). Finally, in a pioneering work, pericycle cells which give rise to lateral roots were captured via laser microdissection (Schnable et al., 2004) and subjected to a proteome analysis (Dembinsky et al., 2007). As a starting point for future cell-type specific analyses, this initial study identified the twenty most abundant soluble proteins of the maize pericycle cell proteome (Dembinsky et al., 2007).

### Root Hairs

Root hairs are tubular extensions of trichoblasts in the epidermis, which significantly increase the absorbing surface of the root and thus the uptake of nutrients (Gilroy and Jones, 2000). In maize, root hair patterning is random and it is not predictable which epidermal cells form root hairs (Dolan, 1996). Several genes control root hair elongation in maize. The *root hair defective* genes *rth3* (Hochholdinger et al., 2008), *rth5* (Nestler et al., 2014), and *rth6* (Li et al., 2015) are functionally linked in the processes of cell wall loosening, cellulose synthesis, and organization of synthesized cellulose, respectively. In contrast, the gene *rth1* (Wen et al., 2005) encodes a SEC3 subunit of the exocyst complex (Hala et al., 2008). To gain a better insight into the root hair proteome, a reference set of 2,573 soluble proteins of maize root hairs of 4-day-old primary roots of the inbred line B73 was identified (Nestler et al., 2011). Root hairs are an ideal model for single cell proteome analyses in maize because they can be easily separated from the primary root after freezing the roots in liquid nitrogen. Among these root hair proteins, homologs of 252 proteins have been associated with root hair development in other species (Nestler et al., 2011). A comparison of the maize root hair reference proteome with those of soybean revealed conserved, but also unique protein functions related to root hairs in these species (Nestler et al., 2011).

## Proteomics of Heterosis Manifestation in Maize Roots

Heterozygous F_1_-hybrids of maize typically perform better than their homozygous genetically distinct parental inbred lines (Hochholdinger and Hoecker, 2007). This phenomenon, known as heterosis can already be observed during early maize primary root development (Hoecker et al., 2006). It has been demonstrated that very young primary roots of hybrids display protein accumulation patterns different from the average of their parental values even before the manifestation of developmental differences in comparison to their parental inbred lines (Hoecker et al., 2008). Among these non-additively accumulated proteins, the functional classes “metabolism” and “disease/defense” were the most abundant (Hoecker et al., 2008). Similar patterns of non-additive protein accumulation were observed in maize embryos of reciprocal hybrids and their parental inbred lines 25 and 35 days after pollination (Marcon et al., 2010). In these embryos, the functional classes “development”, “protein metabolism”, “redox-regulation”, “glycolysis”, and “amino acid metabolism” were most prevalent among non-additively accumulated proteins. In 35-day-old embryos, enzymes related to “glucose metabolism” were significantly upregulated and their expression often even exceeded the expression level of the better parent (Marcon et al., 2010). Finally, the 970 most abundant soluble proteins of 2–4 cm long seminal roots were quantified by label-free LC–MS/MS in a shot-gun approach (Marcon et al., 2013). Consistent with the results of the comparative proteomic dissection of primary roots (Hoecker et al., 2008) and embryos (Marcon et al., 2010), the category “protein metabolism” was also the most prevalent functional class of non-additive proteins in seminal roots (Marcon et al., 2013) when comparing hybrids and their parental inbred lines. In this category, 16 of 17 non-additively accumulated ribosomal proteins displayed high or above better-parent expression in seminal roots (Marcon et al., 2013).

The major findings summarized in Sections “Proteomics of Maize Root System Architecture” and “Proteomics of Heterosis Manifestation in Maize Roots” have been highlighted in **Figure 1**.

## Conclusion

In recent years, numerous studies have surveyed proteomic changes in different root types of maize to understand the complex molecular interactions during root development. Proteome studies are an important tool to complement transcriptome studies because proteins are the biologically active molecules in the cell and there is typically only a moderate correlation between the levels of a transcript and protein encoded by a same gene (e.g., Marcon et al., 2015). This discrepancy can be explained by differences in RNA versus protein stability but also by the fact that one gene can give rise to many proteins by alternative splicing or post-translational modifications. Proteome studies are technically more challenging than transcriptome studies because it is impossible to solubilize all proteins in a single experiment because each tissue contains proteins that range from highly hydrophobic to hydrophilic. In contrast to nucleic acids, proteins cannot be amplified and therefore lowly abundant proteins cannot be detected. Despite these technical limitations it was recently possible to survey tissue specific differences of the primary root proteome and phosphoproteome in high resolution detecting more than 11,000 proteins. With the advent of mass spectrometry, even higher resolution proteome analyses and thus more detailed accounts of proteomic changes during maize root development will become available. While transcriptome analyses are already performed on the level of individual cell-types, such proteome analyses are currently only available for very specific and exposed cell-types that can be easily separated from the rest of the root such as root hair cells (Nestler et al., 2011). With future technological advances of mass spectrometry, the detection of minute amounts of proteins isolated from complex tissues by techniques such as laser microdissection might become available.

## Author Contributions

All authors contributed to the writing of this minireview. JB drew the maize seedling displayed in **Figure 1**.

## Conflict of Interest Statement

The authors declare that the research was conducted in the absence of any commercial or financial relationships that could be construed as a potential conflict of interest.
